# Identification and phylogenetic analyses of VASt, an uncharacterized protein domain associated with lipid-binding domains in Eukaryotes

**DOI:** 10.1186/1471-2105-15-222

**Published:** 2014-06-26

**Authors:** Mehdi Khafif, Ludovic Cottret, Claudine Balagué, Sylvain Raffaele

**Affiliations:** 1 INRA, Laboratoire des Interactions Plantes-Microorganismes (LIPM), UMR441, 24 Chemin de Borde Rouge – Auzeville, CS52627, F31326 Castanet Tolosan Cedex, France; 2 CNRS, Laboratoire des Interactions Plantes-Microorganismes (LIPM), UMR2594, 24 Chemin de Borde Rouge – Auzeville, CS52627, F31326 Castanet Tolosan Cedex, France

**Keywords:** VASt, VAD1, Protein domain, Programmed cell death, GRAM domain, Bet v1-like

## Abstract

**Background:**

Several regulators of programmed cell death (PCD) in plants encode proteins with putative lipid-binding domains. Among them, VAD1 is a regulator of PCD propagation harboring a GRAM putative lipid-binding domain. However the function of VAD1 at the subcellular level is unknown and the domain architecture of VAD1 has not been analyzed in details.

**Results:**

We analyzed sequence conservation across the plant kingdom in the VAD1 protein and identified an uncharacterized VASt (VAD1 Analog of StAR-related lipid transfer) domain. Using profile hidden Markov models (profile HMMs) and phylogenetic analysis we found that this domain is conserved among eukaryotes and generally associates with various lipid-binding domains. Proteins containing both a GRAM and a VASt domain include notably the yeast Ysp2 cell death regulator and numerous uncharacterized proteins. Using structure-based phylogeny, we found that the VASt domain is structurally related to Bet v1-like domains.

**Conclusion:**

We identified a novel protein domain ubiquitous in Eukaryotic genomes and belonging to the Bet v1-like superfamily. Our findings open perspectives for the functional analysis of VASt-containing proteins and the characterization of novel mechanisms regulating PCD.

## Background

Protein domain predictions are a starting point for a range of functional analyses and can either newly predict or further refine functional predictions [1]. Indeed, domains form structural, evolutionary and functional units of proteins [2]. The combination and order of domains in a protein is frequently considered as a fundamental level of protein functional complexity. The majority of proteins is composed of multidomain proteins and the domain composition of multidomain proteins is critical for their specialized functions [3]. Furthermore, domain combinations are not random, which may indicate functional cooperation [4].

Plant “lesion mimic mutants” (LMMs) show spontaneous necrotic lesion resembling the so-called Hypersensitive Response (HR), a form of programmed cell death associated with plant defense [5,6]. The *Arabidopsis thaliana vad1* (*vascular associated death1*) mutant is a LMM altered in a negative regulator of PCD and defense responses harboring a GRAM domain predicted to bind lipids [7,8]. Contrary to most LMM genes characterized to date, *VAD1* is expected to control the cell-to-cell propagation of PCD instead of its initiation [5]. GRAM is a ~70 amino-acids domain predicted to mediate intracellular protein binding or lipid binding during membrane-associated processes [9]. This domain is related to the PH domains and is found in animal glucosyltransferases, Rab-like GTPase activators, myotubularins and other membrane-associated proteins. The GRAM domain of human myotubularins is able to bind phospholipids and is involved in membrane signaling [10-12], but its exact function often remains enigmatic [13].The presence of a GRAM domain in VAD1 protein suggests that lipid binding could be required for VAD1 function, but the role of VAD1 at the subcellular level is currently unknown.

Remarkably, a significant proportion of plant LMMs show mutations in genes associated with lipid biosynthesis and homeostasis [14-17]. This notably concerns sphingolipid metabolism: the *acd5* and *acd11* mutants carry mutations in a ceramide kinase and a putative sphingosine transfer protein, respectively [15,16]. Conversely, *ERH1* is a positive regulator of the HR encoding a functional inositolphosphoryl-ceramide (IPC) synthase which converts ceramide to IPC [18]. Furthermore, *EDR2* was isolated as a negative regulator of PCD and defense responses encoding a multi-domain protein featuring a DUF1336 domain, a PH domain and a START domain [17,19]. Like CERT PH domain, EDR2 PH domain preferentially binds to PI4P [19]. Nevertheless, the mechanisms by which lipid-binding domain containing proteins regulate PCD in plants are largely unknown.

Here, we analyzed sequence conservation in the VAD1 family and identified an uncharacterized conserved domain we designated as VASt (*V*AD1 *A*nalog of *St*AR-related lipid transfer). Using sequence- and structure-based phylogenetic analyses we demonstrate that this domain is present in all major eukaryotic lineages but no molecular function has been assigned to it. VASt is related to Bet v1-like, a superfamily including lipid- and hormone-binding domains. The VASt domain will be referred to with accession number PF16016 in release 28.0 of the Pfam database [20]. These findings open new perspectives for the functional analysis of VASt-domain containing proteins such as *A. thaliana* VAD1, yeast YSP2 and human GRAM1A, B and C.

## Results

### Proteins in the VAD1 family contain an uncharacterized conserved domain

**Figure 1:**
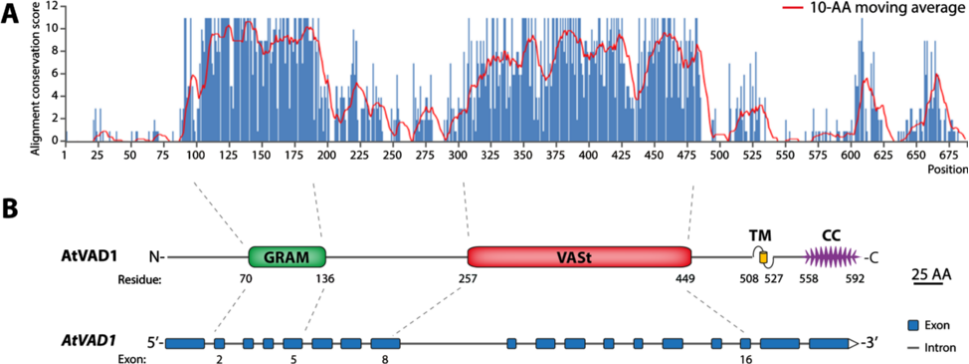
**Identification of an uncharacterized conserved protein domain in AtVAD1 homologs. (A)** Amino acid conservation score along an alignment of AtVAD1 13 homologs. The histogram shows conservation score for each position of the alignment; the red line shows 10-AA moving average. **(B)***Top*: AtVAD1 protein schematic diagram with known domains (GRAM, glucosyltransferases, Rab-like GTPase activators, myotubularins; VASt, VAD1 analog of START; TM, transmembrane helix; and CC, coiled-coil). Residue number corresponds to amino acid position of AtVAD1 domains. *Bottom*: Genomic organization of *AtVAD1* gene showing intron/exon structure. AA, amino acids.

To get insights into VAD1 putative biochemical function, we analyzed protein sequence conservation among VAD1 homologs. First, to identify VAD1 homologs, we used the full length sequence of VAD1 protein in stringent phmmer searches against the Uniprot database. We identified 13 AtVAD1 homologs with e-value below 1e^-100^ across twelve angiosperm plant species, including monocots (*Brachypodium distachyon*, *Musa acuminata*, *Oryza brachyantha*, *Oryza sativa*, *Setaria italica*, *Sorghum bicolor*) and eudicots (*Arabidopsis lyrata*, *Glycine max*, *Ricinus communis*, *Solanum lycopersicum*, *Vitis vinifera*). In this stringent approach, all species showed a single VAD1 copy except *G. max* that had three copies. The retrieved homologs showed at least 55% identity and all contained a clearly identified GRAM domain. To identify conserved regions in these 14 sequences, we aligned them using the Multiple Sequence Alignment (MSA) tool MAFFT [21] and we plotted the consensus conservation score along the alignment using a ten Amino-Acids (AA) sliding window (Figure 1A). Two major conserved regions were clearly apparent. The first one spanning positions 80 to 200 in the alignment, and the second spanning positions 300 to 480. To characterize and precisely delimit VAD1 conserved regions we mapped VAD1 gene and protein annotations onto the conservation plot (Figure 1B). The N-terminal conserved region (position 80–200) overlapped largely with the predicted GRAM domain (position 116–182). The second conserved region (position 300–480) corresponded to an uncharacterized domain of approximately 190 AA. This domain is encoded by a region spanning from *AtVAD1* exon 8 to exon 16. We therefore set the limits of this uncharacterized domain at positions 257 and 449, for a total length of 193 AA (Figure 1B). Based on further characterization described hereafter, we designated this domain as the VASt (*V*AD1 *A*nalog of *ST*ART) domain. A close up view on the MSA of the VASt domain revealed a high degree of conservation among plant homologs, with an average 70.5% identity over the VASt domain (Additional file 1: Figure S1, Additional file 2). Since no annotation could be mapped onto the VASt domain, it represents a yet uncharacterized protein domain highly conserved in plants.

### The VASt domain is conserved among Eukaryotes

**Figure 2:**
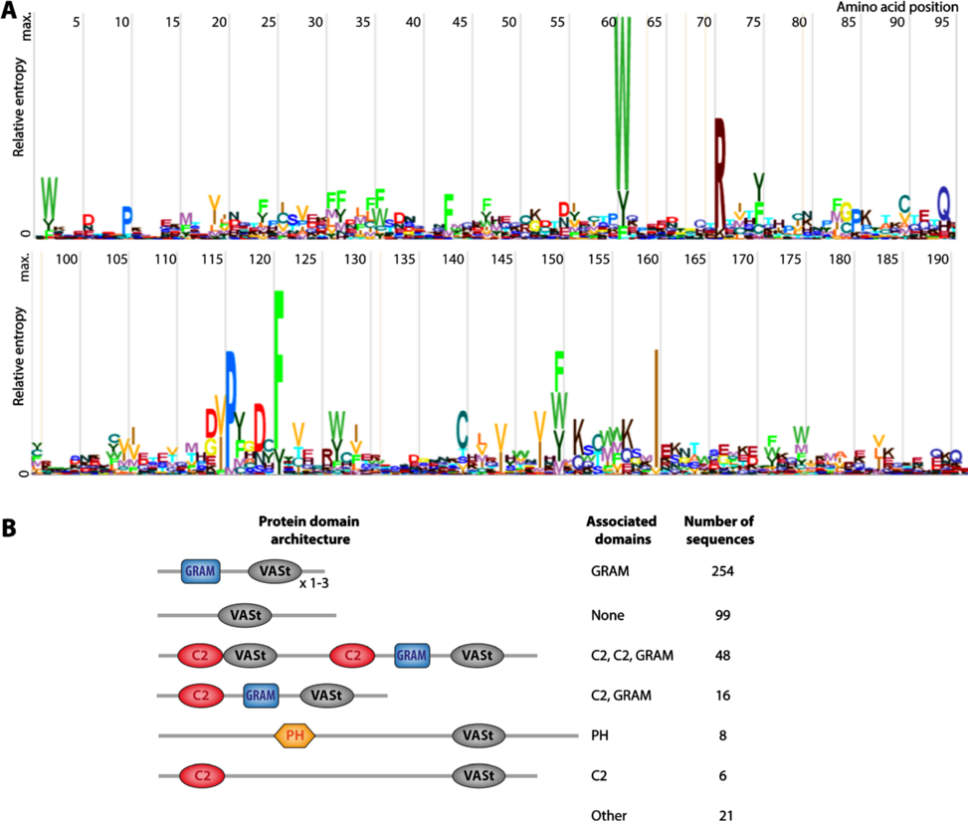
**Amino acid conservation in the VASt domain and domain architecture of VASt-containing proteins. (A)** HMM logo of the 193 AA VASt domain. Relative entropy is measured in bits. High entropy values indicate a high degree of certainty that the corresponding amino acid is present in orthologs domain. AA are colored to represent structural or functional similarity. The width of the amino acid positions indicates the probability of insertions (wider) and deletions (narrower). **(B)**. Domain architecture of VASt-containing proteins. Schematic representation of the proteins retrieved after 3 iterations using jackhmmer tool.

Since none of AtVAD1 homologs had characterized biochemical functions, we extended our search of related protein domains using Hidden Markov Models (HMM) to get insights into the VASt domain putative function and evolution. For this, we built a HMM using the 13 plant homologs of AtVAD1 VASt domain. To highlight important sequence motifs in this HMM, we examined the corresponding sequence logo (Figure 2A). Amino acids W61, R71, P116, F121 and I160 were strongly conserved in the model, suggesting an important contribution of these residues to the protein function. To identify protein domains related to the VASt domain, we searched the Uniprot database with the VASt HMM model. After three iterations of jackhammer search, we retrieved 452 hits distributed exclusively in eukaryotic proteins, including yeasts and other Fungi, Oomycetes, Mammals, and Plants (Additional file 3). Most of the identified proteins contained one copy of the VASt domain (~85.8%), with a few containing two or more VASt domains (Figure 2B). As little as ~21.9% of the protein hits contained VASt as the only domain identified. The VASt domain is frequently associated with lipid binding domains such as GRAM (in 70.3% of the hits), C2 and Pex24p domains, suggesting a functional link between VASt and lipid-binding domains. Among proteins related to VAD1 retrieved by our HMM search was YSP2, a major cell death regulator in Yeast [22]. However, there was no protein for which a biochemical function has been described.

### Multiple domain combinations contributed to the diversification of VASt-containing proteins

**Figure 3:**
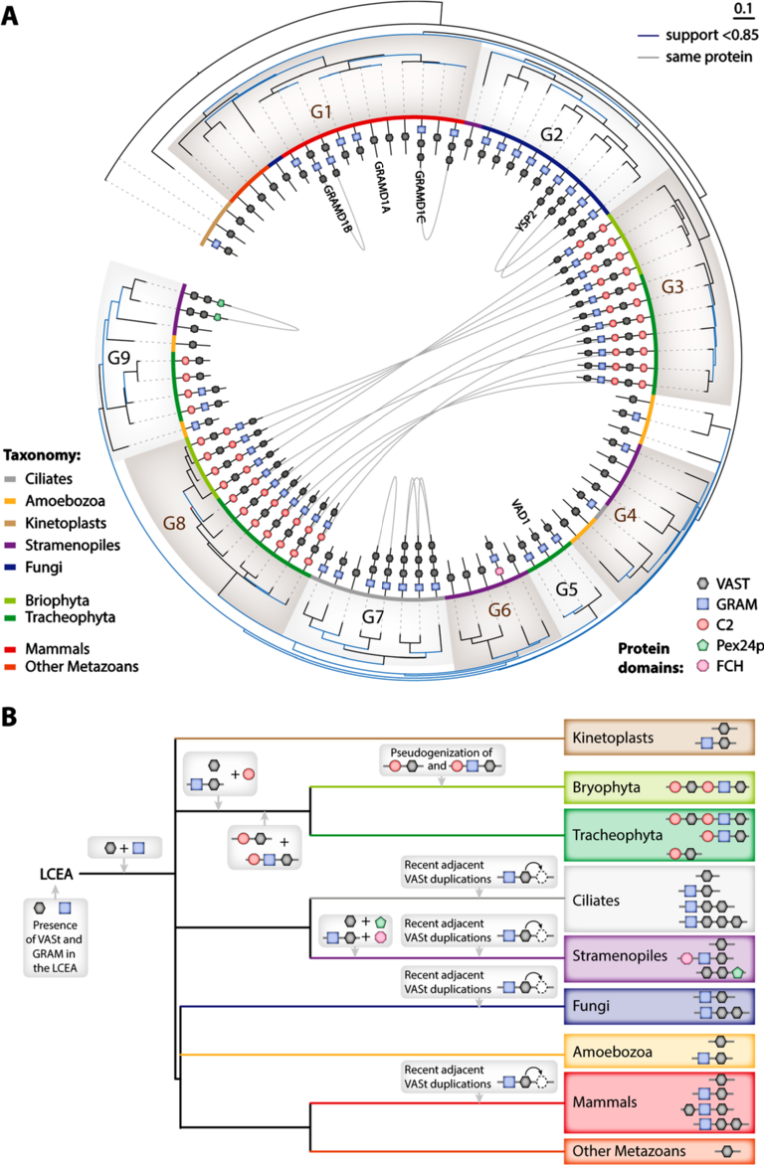
**Phylogeny and evolutionary history of the VASt domain. (A)** Maximum likelihood phylogenetic tree of 85 VASt protein domains from 17 fully-sequenced species representative of major Eukaryotic lineages. Multiple copies of VASt found in a single protein are linked with central connectors. Protein Domain architecture and taxonomy are shown along branches. Nine phylogenetic groups (G1 to G9) are highlighted. Proteins cited in the main text are labeled along branches. **(B)** Proposed scenario for the evolution of VASt-containing proteins. Domain symbols are as in **A**. LCEA, last common Eukaryote ancestor.

To document the evolution of the VASt domain, we examined the phylogenetic relations of VASt domains in 17 fully sequenced species representing all major Eukaryotic lineages (see Methods), corresponding to a total of 85 protein sequences (after redundancy and incomplete sequence filtering). (Figure 3, Additional file 4, Additional file 5, Additional file 6). Sequences clustered into nine groups defined by their taxonomic range and domain organization. To highlight the phylogenetic relationship between copies of the VASt domain present in a single protein (clades 1, 2, 4 and 5), we have connected together VASt copies present in the same protein (Figure 3A). Group 1 gathered sequences from mammals and other metazoans. In this group, the VASt domain is found either (i) alone, (ii) associated with a GRAM domain or (iii) associated with another VASt domain. The two copies of VASt in GRAM-VASt-VASt proteins clustered in the same clade indicating that the duplication of VASt domain is recent. This group included human GRAMD1A, GRAMD1B and GRAMD1C proteins. Group 2 exclusively consisted in sequences from fungi, with the same domain structures as found in Group 1. Group 2 contained the yeast YSP2 protein. Group 3 and 8 contained N-terminal and C-terminal VASt domains respectively of proteins with a C2-VASt-(C2)-GRAM-VASt domain architecture. Group 3 and 8 are restricted to plants, including the moss *Physcomitrella patens*, and phylogenetically distant, suggesting that duplication of the VASt domain early in land plant evolution allowed the divergence of two VASt copies in these proteins. Group 4 contained proteins from Stramenopiles, Ciliates and Amoebozoa, with either a VASt domain alone or a GRAM-VASt architecture. Group 5 contained exclusively plant sequences with a GRAM-VASt structure, include *A. thaliana* VAD1. Group 6 contained exclusively Stramenopile sequences with either a VASt domain alone or a FCH-GRAM-VASt architecture. Group 7 contained exclusively Ciliate sequences comprising either a VASt domain alone, or GRAM domain with one to three copies of VASt. Finally, Group 9 contained sequences from Amoebozoa, Tracheophyta (vascular plants) and Stramenopiles with diverse domain architectures: (i) VASt alone, (ii) GRAM-VASt, (iii) C2-GRAM-VASt or (iv) VASt-VASt-Pex24p. Groups 3, 5, 8 and 9 show support value of 1.0 suggesting that the function of VASt domains from these groups could have diverged, and that this divergence could be essentially driven by the association with the C2 and the GRAM domains.

Next, we attempted to reconstruct the evolutionary history of domain combinations in VASt-containing proteins (Figure 3B). Similar to GRAM [13], the VASt domain would originate from the last common eukaryote ancestor (LCEA). Since GRAM and VASt domains are associated in nearly all eukaryotic lineages the GRAM-VASt combination probably dates back from the LCEA. Sequences harboring a VASt domain alone were also found in all lineages except Plants and Fungi, suggesting that a copy of the ancestral VASt domain gene has been maintained in most phyla. Alternatively, the GRAM domain could have been lost from a putative GRAM-VASt ancestor in several lineages. Adjacent VASt duplications within a single protein are observed in Ciliates, Stramenopiles, Fungi and Mammals that probably arose recently, judging from high sequence similarity between adjacent copies. Consistent with [13], VASt association with both GRAM and C2 appeared Plant-specific. A parsimonious scenario for the emergence of the complex C2-VASt-C2-GRAM-VASt domain architecture specific to Plants could be the combination of a C2 domain with ancestral VASt alone and GRAM-VASt proteins, followed by the fusion of a C2-VASt and a C2-GRAM-VASt module early in the evolution of Plants (Figure 3B). The C2-VASt and C2-GRAM-VASt modules could have been maintained in vascular plants but not in mosses. Alternatively, a VASt-GRAM-VASt fusion could have emerged in an ancestral Eukaryotic lineage (it has been maintained in Mammals), combined with two C2 domains in plants, then C2-VASt and C2-GRAM-VASt could have emerged from the split of a C2-VASt-C2-GRAM-VASt plant ancestor in vascular plants but not in mosses. The association of VASt with Pex24p or FCH domains seems to be innovations from the Oomycete lineage. In addition to data presented in Figure 3, proteins with a PH-VASt architecture were found in some fungal species.

### Homology modeling of the VASt domain 3D structure

**Figure 4:**
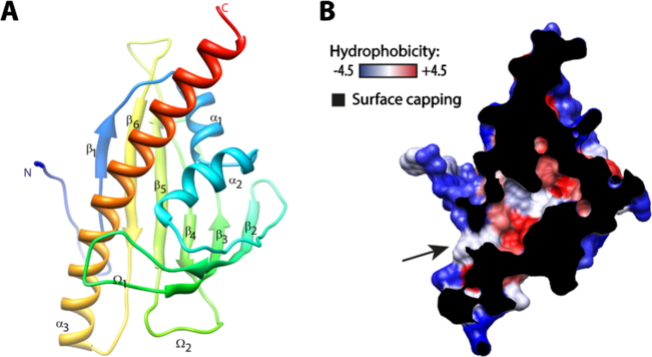
**Homology modeling of the VASt domain 3D structure. (A)** Ribbon diagram representation of the predicted 3D structure of AtVAD1 VASt domain, colored from blue (N-terminus) to red (C-terminus). Secondary structure elements are numbered from N to C terminus. **(B)** The VASt domain harbors a large hydrophobic cavity. Surface of AtVAD1 VASt model colored according to residue hydrophobicity. Capping surface is shown in black, the entrance of the hydrophobic cavity is marked by an arrow.

Classical sequence-based phylogeny did not allow identifying protein domains of known function related to VASt. Structure-based phylogenetic network inference may be used to improve the resolution of deep evolutionary relationships and assist in inference of protein function [23]. To analyze relationships between VASt and protein domains of known three-dimensional structure, we conducted a structure-based clustering of AtVAD1 VASt domain and its closest analogs. First, to obtain atomic coordinates of a 3D model for AtVAD1 VASt domain, we submitted its 193 AA sequence to the homology and threading structure prediction server I-TASSER [24]. The best model (Additional file 7) showed a two-layer sandwich alpha beta fold (CATH 3.30, also called “helix grip fold”, [25]) containing three alpha helices (α1 to 3), six beta-sheets (β1 to 6) and two loops (Ω1 and 2) numbered from N to C terminus (Figure 4A). This model had a C-score of -1.41indicating that quality predictions can be estimated with more than 90% confidence [24], and expected TM-score of 0.54 suggesting a correct topology. Eight of the top 10 threading alignments had normalized Z-score higher than 1, thus accuracy of the model is expected to be high [26]. The predicted VASt model encompasses a large hydrophobic cavity delimited by sheets β2, 3 and 4, loop Ω1 and helices α2 and 3 (Figure 4B).

### Structure-based phylogeny reveals relationships between the VASt domain and Bet v1-like domains

**Figure 5:**
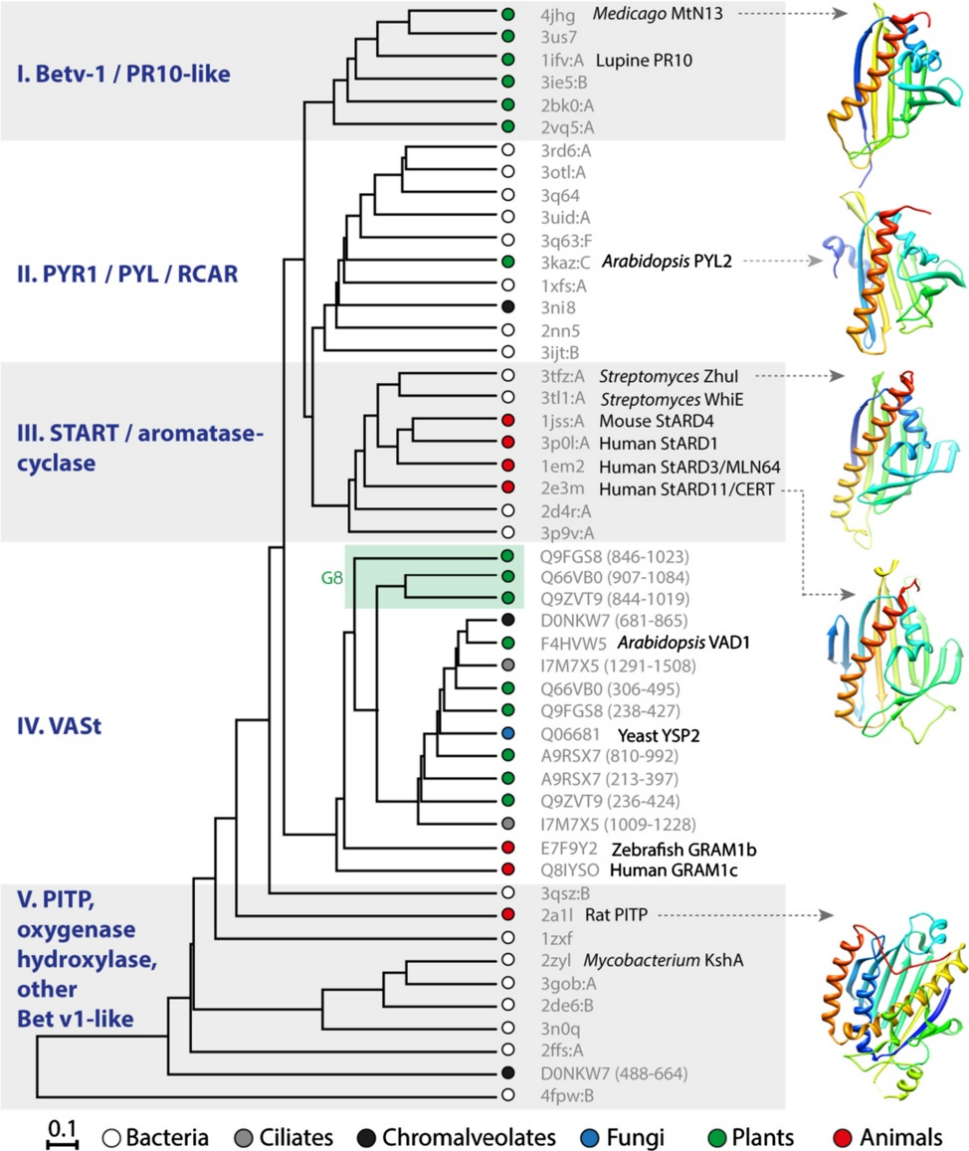
**Structure-based phylogeny reveals relationships between the VASt domain and Bet v1-like domains.** Structure-based neighbor joining tree including 16 VASt domains and 33 structural analogs identified by fold recognition searches. Representative proteins are labeled along branches and representative structures shown as ribbons colored from blue (N-terminus) to red (C-terminus). Terminal nodes are colored according to taxonomic distribution. Five structural groups (I to V) are highlighted, VASt domains from phylogenetic group 8 (G8) are also indicated. Bet v1, *Betula verrucosa* pollen antigen 1; PR-10, pathogenesis-related 10; PITP, phosphatidylinositol transfer protein; StAR, steroidogenic acute regulatory protein; StARD, STAR domain protein; START, StAR-related lipid-transfer; PYR1, pyrabactin resistance 1; PYL, PYR1-like; RCAR, Regulatory components of abscisic acid receptors.

**Figure 6:**
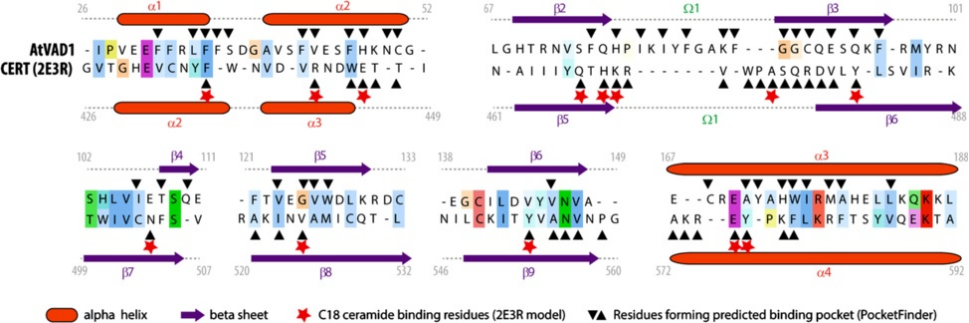
**Sequence alignment of regions corresponding to the ligand binding pocket in AtVAD1 VASt domain and CERT.** Structural annotations are indicated above and below sequences for AtVAD1 and CERT respectively.

To investigate structural relationships between AtVAD1 VASt domain and its closest analogs, we conducted a structure-based tree inference analysis including AtVAD1 VASt domain, predicted three-dimensional structure for 15 VASt homologs, and the top structural analogs retrieved by fold recognition searches. The best structural analogs retrieved by I-TASSER and NCBI Vector Alignment Search Tool fold recognition searches were human MLN64 (Metastatic axillary lymph node protein 64) STAR-related lipid transport domain [PDB:1EM2] and *Streptomyces* ZhuI polyketide aromatase/cyclase [PDB:3TFZ]. Close analogs also included human CERT ceramide trafficking protein [PDB:2E3M] and *Arabidopsis thaliana* PYL2 ABA receptor [PDB:3KDI]. Gene Ontology terms associated with AtVAD1 VASt domain based on the 3D model included hormone binding [GO:0042562], isoprenoid binding [GO:0019840] and monocarboxylic acid binding [GO:0033293]. To build a structure-based phylogenetic tree, we modeled the three-dimensional structure of 15 VASt domains using AtVAD1 VASt as a template, and searched for AtVAD1 VASt closest structural analogs in the medium redundancy subset of the Molecular Modeling database (Additional file 8). We next performed a multiple structure alignment, calculated normalized pairwise RMSD distances for aligned Cα atoms (Additional file 9), and used this distance matrix to produce a neighbor-joining tree (Additional file 10). Structures clustered into five major groups (Figure 5). Group I contained plant Bet v1 phytohormone-binding proteins and pathogenesis-related (PR) 10-like proteins. Group II contained *Arabidopsis thaliana* PYL2 abscisic acid (ABA) receptor, belonging to the Pyrabactin resistance 1 (PYR1)/PYR1-like (PYL)/Regulatory components of ABA receptors (RCAR) family and uncharacterized bacterial proteins. Group III contained mammalian START proteins binding sterols and sphingolipids, and *Streptomyces* aromatase-cyclases. Group IV contained VASt-domains. Finally Group V contained mammalian phosphatidylinositol transfer protein (PITP), bacterial oxygenase and hydrolases, and other Bet v1-like domains. With the exception of the N-terminal VASt domain of *Phytophthora infestans* predicted protein PITG_12663 [Uniprot:D0NKW7], all VASt domains clustered together into structural group IV. A separate sub-group within structural group IV was formed by VASt domains from sequence-based phylogenetic group 8, supporting a possible functional divergence. This analysis revealed that VASt domains are structurally related to Bet v1-like domains [Pfam:CL0209] known to bind bulky hydrophobic ligands such as phytohormones, lipids and polyketides. Association with lipid-binding domains in large multidomain proteins is typical for START and VASt domains.To further test whether AtVAD1 VASt domain and the well-characterized START domain of the CERT protein could be evolutionary related, we closely examined the ligand-binding pockets of these two proteins. For this, we performed a structural alignment of AtVAD1 VASt and CERT START domain models. The structural alignment of AtVAD1 VASt domain and CERT START domain highlighted a good conservation of secondary structure elements lining the substrate binding pocket (Figure 6). Although the overall sequence conservation between the two proteins is limited, residues binding to C18 ceramide in CERT showed conservation or similar environments in AtVAD1 VASt domain. These observations are consistent with hidden homology between VASt and Bet v1-like domains. Alternatively, the analogy between VASt and Bet v1-like domains could result from convergent evolution. Testing the importance of the predicted ligand-binding pocket residues for VASt function could help discriminate between these hypotheses.

## Discussion

We report the identification of the VASt protein domain in the VAD1 plant cell death regulator. This domain is conserved across eukaryotes and is structurally related to Bet v1-like domains, including START lipid-binding domains. The predicted structure of VAD1 VASt domain is consistent with a function in binding large hydrophobic ligands. Our findings open new perspectives for the analysis of functions of the VASt domain associated with the GRAM, C2 and PH lipid-binding domains and the characterization of novel mechanisms regulating PCD in plants.

What is the physiological role of VASt-containing proteins? Most of the proteins containing VASt domain have no characterized function to date. The *A. thaliana* VAD1 is the only exception in plants. In the context of pathogen attack, controlled programmed cell death (PCD) is one of the prevailing plant defense responses, allowing confinement of the pathogen locally in dead cells. The *vad1* mutant exhibits spontaneous PCD lesions initiated in cells surrounding vascular tissue progressively expand to the whole leaf, hence its classification as “propagation lesion mimic mutant” [7]. This phenotype suggests that *vad1* is impaired in the control of cell-to-cell propagation of PCD, involving a yet unknown mechanism.Amiodarone is a Ca^2+^ channel-targeted drug inducing apoptosis mediated by reactive oxygen species (ROS), *via* the same pathway as natural pheromones [27]. Genetic screens have revealed the function of the YSP2 (Yeast Suicide Protein 2) in enhancing survival after amiodarone treatment. YSP2 is a mitochondrial membrane protein involved in mitochondrial fragmentation, probably acting downstream of ROS production triggered by intracellular acidification [22]. YSP2 harbors a GRAM and a VASt domain but its molecular function is unknown. In human, a polymorphism in the GRAMD1B gene has been associated with susceptibility to chronic lymphocytic leukemia [28]. Recently, a whole genome modified-siRNA screen identified GRAMD1B as a protein associated with chemoresistance in epithelial ovarian cancer (OvCa) cells. Consistent with the view that acquired chemoresistance is a major contributor to patient mortality from OvCa, reducing GRAMD1B expression increased overall survival in OvCa patients and decreased tumour burden in mouse models [29]. GRAMD1C has been identified as part of a quantitative trait locus associated with hepatic iron overload in mice, but its function has not been validated [30]. The molecular function of GRAMD1 proteins has not been investigated but their association with several disorders supports the relevance of VASt-containing proteins for cell integrity. What may be the signal(s) associated to VAD1 that mediate propagation of PCD? The VASt domain is related to domains from the Bet v1-like superfamily [Pfam:CL0209] that bind large hydrophobic ligands such as lipids, hormones and antibiotics [31]. In the Bet v1-like superfamily, PR-10, Bet v1 and PYR/PYL/RCAR domains (Figure 5, groups I and II) typically bind phytohormones such as brassinosteroids, cytokinins and abscisic acid [32-34]. Some other Bet v1-like domains (Figure 5, groups III and V) bind secondary metabolites such as flavonoids, polyketides and various antibiotics [31,35]. These ligands are diffusible molecules that could act as intercellular signals regulated by VAD1. Domains belonging to the START subfamily of Bet v1-like domains bind lipids such as sterols and sphingolipids [31,36]. In animal cells, intercellular transport of sterols and sphingolipids is mainly mediated by non-vesicular transport *via* the action of dedicated lipid transport proteins (LTPs) or *via* spontaneous lipid exchange [37]. Phytohormone- and secondary metabolite-binding proteins in the Bet v1-like superfamily often function as single-domain proteins, or multimers of single domain-proteins, whereas START and VASt-containing proteins are generally large multidomain proteins. Notably, the mammalian CERT and *Arabidopsis* VAD1 proteins share a common domain structure involving a PH superfamily domain (PH and GRAM respectively) and a Bet v1-like superfamily domain (START and VASt respectively). Cooperation between the PH and START domains in CERT is critical for its function as a ceramide transport protein [38]. Ceramides and other sphingolipids are important regulators of cell death programs in animals and plants [39,40]. VAD1 may therefore sense or transport lipids to modulate cell death signals intercellularly.

What is the evolutionary history of the VASt domain? Our sequence- and structure-based phylogenetic analyses suggest that the VASt domain evolved from a primordial Bet v1-related protein that existed in the last universal common ancestor, and emerged with the divergence of Eukaryotes. Alternatively, the analogous structure of VASt and Bet v1-like domains could result from convergent evolution. By contrast to the PR10-like subfamily of Bet v1 domains, the VASt domain is conserved across all major Eukaryotic lineages, and therefore probably serves a function relatively conserved across all Eukaryotes. Radauer *et al*. proposed that the primordial Bet v1 protein would bind lipids, and would have evolved by addition of secondary structural elements or fusion to other domains into multi-domain proteins [31]. Our results suggest that the VASt domain has been associated with the GRAM domain very early in the history of Eukaryotes, and was later combined with C2 domains in Plants and with Pex24p domains in Oomycetes. The 3D model we obtained for VAD1 VASt domain features a long loop connecting helix α2 and sheet β2, instead of a beta-sheet in typical Bet v1 domains, leading to a β-α2-β5-α instead of β-α2-β6-α secondary structure arrangement. VASt domains of Plants span across four phylogenetic groups (Group 3, 5, 8 and 9, Figure 3), suggesting the emergence of novel adaptations in the Plant kingdom, that may either reflect the evolution of new catalytic activities or the adaptation to plant-specific ligand(s).

## Conclusions

Local variations in membrane protein and lipid composition create subcellular compartments with diverse physico-chemical properties. Such local variations in lipid and protein content may be critical for defining the specific structure membrane compartments and flagging them for the addressing of proteins and other signals [41-43]. The trafficking routes between membrane compartments, and the proteins implicated, are just starting to be uncovered. Our analyses revealed the VASt domain as a member of the Bet v1-like superfamily predominantly associated with lipid binding domains such as GRAM, C2 and PH domains. This finding opens new perspectives for molecular and genetics studies of the function and regulation of VASt domain containing proteins.

## Methods

### Identification of *VAD1* homologs and conservation analysis in plants

The VAD1 protein sequence [Uniprot: F4HVW5] was used as a query for a profile Hidden Markov Model (HMM) search with phmmer [44] against the Uniprot database using Blosum62 matrix. Hits with e-value 1e^-100^ or less were selected and manually curated resulting in the identification of 13 homologs, exclusively from plants. Sequences were aligned using MAFFT version 7 [21] with default parameters. Amino acids conservation score, calculated according to [45], was plotted as a moving average using a sliding window approach with window size 10 and steps of size 1.Protein sequence features retrieved through the phmmer search were manually mapped along the alignment. AtVAD1 gene model (At1g02120.1) was retrieved from TAIR (http://www.arabidopsis.org) and mapped on the protein alignment using GeneWise (http://www.ebi.ac.uk/Tools/psa/genewise/) with default parameters.

### Conservation and phylogenetic analyses of VAD1 VASt domain

The precise boundaries of the newly identified VASt domain were set based on a conservation score >4 among plant AtVAD1 homologs and to include well-conserved N-terminal residues F1, D7 and P11, delimiting a 193 amino acids domain. The ungapped alignment of the 193 amino acids domain of VAD1 VASt domain with its 13 homologs was used as entry for HMM searches with jackhmmer [44] using Blosum90 matrix against NR database, with cut off e-value of 1e^-10^. The final list of proteins containing VASt domains was obtained after 3 iterations of jackhammer search using all hits from previous iteration as a seed. Sequence logo of the 452 VASt domains alignment was done with LOGOMAT-M [46]. To built the VASt domains phylogenetic tree, we selected all hits from 17 fully sequenced species representative of all major Eukaryotic lineages as follows: *Homo sapiens* and *Mus musculus* (Mammals), *Caenorhabditis elegans* and *Drosophila melanogaster* (Other Metazoans), *Arabidopsis thaliana* and *Oryza sativa* (Tracheophyta), *Physcomitrella patens* (Briophyta), *Saccharomyces cerevisiae*, *Aspergillus nidulans*, *Neurospora crassa* and *Ustilago maydis* (Fungi), *Phytophthora infestans* and *Thalassiosira pseudonana* (Stramenopiles), *Entamoeba histolytica* and *Dictyostelium discoideum* (Amoebozoa), *Leishmania major* (Kinetoplasts), *Tetrahymena thermophila* (Ciliates). After removing incomplete sequences and redundant sequences with CD-HIT [47], 85 sequences were aligned using MAFFT version 7 [21]. The alignment was automatically curated using TrimAI [48] to keep 125 positions out of 341. Selection of LG + I + G + F as best-fit models with alpha value 2.108 for omega distribution was performed in ProtTest2 [49]. A phylogenetic tree showing branch support values as aLRT SH-like test was generated using PhyML 3.0 [50] and visualized using iTOL [51].

### AtVAD1 VASt domain 3D structure modeling and structure-based clustering

The 3D structure of AtVAD1 (At1g02120) VASt domain (residues 257 to 449) was predicted with I-TASSER web server (http://zhanglab.ccmb.med.umich.edu/I-TASSER/). For structural tree inference, we first searched for AtVAD1 VASt domain structural analogs using the Vector Alignment Search Tool server [52]. Forty six analogs aligned over 100 amino-acids or more were retrieved. Second, we predicted the three dimensional of 15 VASt domains with Modeller v9.12 [53] using AtVAD1 VASt domain as a template. A structural alignment of AtVAD1 VASt domain, 15 other VASt domain structures and 46 structural analogs was generated with Mustang-MR [54]. Pairwise root-mean-square deviation (RMSD) between Cα atoms were normalized as 100*RMSD/number of aligned residues, and used to build the distance matrix for tree inference. The tree was constructed using Fitch-Margoliash method in Phylip [55] with power 2.0, and a number of terminal branches connecting uncharacterized proteins were manually removed for clarity, resulting in a distance tree with 49 structures. All protein structures were rendered with UCSF Chimera [56].

### Availability of supporting data

The original version of the tree shown in Figure 3A can be accessed at http://itol.embl.de/shared/lipm_bioinfo under the project ‘VAST’. All other supporting data associated with this manuscript are included as additional files.

## Competing interests

The authors declare no conflict of interest

## Authors’ contributions

Conceived and designed the analyses: MK, LC, SR. Performed and interpreted the analyses: MK, LC, CB, SR. Wrote the paper: MK, CB, SR. All authors read and approved the manuscript.

## Supplementary Material

Additional file 1: Figure S1 Multiple sequence alignment of VASt domains from AtVAD1 and its 12 closets plant homologs. Positions showing > 70% identity are highlighted in blue. Click here for file

Additional file 2 Multiple alignment file in .FASTA format of AtVAD1 and its 12 closest homologs. Click here for file

Additional file 3 Table of the 452 proteins retrieved from Uniprot after HMM search using the model built from 14 VAD1 homologs as bait. Click here for file

Additional file 4 Multiple alignment file in .FASTA format of 85 VAST domains. Click here for file

Additional file 5 **Multiple alignment file in .FASTA format of 85 VAST domains after clean-up by trimAI, used to generate the tree shown in Figure** 3A. Click here for file

Additional file 6 Phylogenetic tree of 85 VASt-containing proteins from 17 fully-sequenced genomes in newick format. Click here for file

Additional file 7 **Atomic coordinates of AtVAD1 VASt domain best model obtained *via *** **modeling by I-TASSER in .pdb format.** Click here for file

Additional file 8 **List of AtVAD1 VASt domain closest structural analogs identified in the medium redundancy subset of the Molecular Modeling database using NCBI Vector Alignment Search Tool.** Only structures that aligned over 100 residues or more were included in further analyses. Click here for file

Additional file 9 Pairwise RMSD matrix used for the generation of the structure-based neighbor-joining tree of VASt domain analogs in phylip format. Click here for file

Additional file 10 Structure based neighbor-joining tree of VASt domain analogs in newick format. Click here for file
