# Supplementary material for: Identification and phylogenetic analyses of VASt, an uncharacterized protein domain associated with lipid-binding domains in Eukaryotes
Source: BMC Bioinformatics. 2014 Jun 26;15:222. doi: 10.1186/1471-2105-15-222 (PMC4082322; doi:10.1186/1471-2105-15-222)
Supplement: Additional file 1: Figure S1 — Multiple sequence alignment of VASt domains from AtVAD1 and its 12 closets plant homologs. Positions showing > 70% identity are highlighted in blue. [file 1471-2105-15-222-S1.pdf]

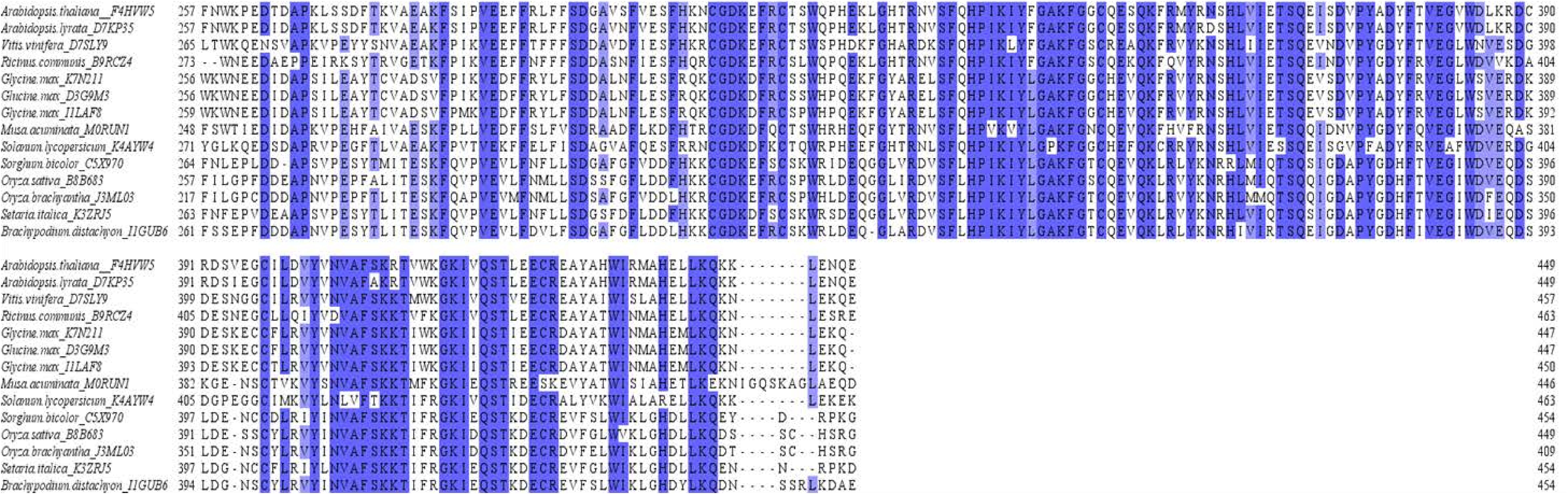

Arabidopsis.thaliana__F4HVW5
Arabidopsis.lyrata_D7KP35
Vitis.vinifera_D7SLY9
Ricinus.communis_B9RCZ4
Glycine.max_K7N211
Glucine.max_D3G9M3
Glycine.max_I1LAF8
Musa.acuminata_M0RUN1
Solanum.lycopersicum_K4AYW4
Sorghum.bicolor_C5X970
Oryza.sativa_B8B683
Oryza.brachyantha_J3ML03
Setaria.italica_K3ZRJ5
Brachypodium.distachyon_I1GUB6
